# Potentiating the Immune Responses of HBsAg-VLP Vaccine Using a Polyphosphoester-Based Cationic Polymer Adjuvant

**DOI:** 10.1021/acsami.3c07491

**Published:** 2023-10-10

**Authors:** Xuhan Liu, Yifan Liu, Xiaoyu Yang, Xinyu Lu, Xiao-Ning Xu, Jiancheng Zhang, Rongjun Chen

**Affiliations:** † Department of Chemical Engineering, Imperial College London, South Kensington Campus, London SW7 2AZ, U.K.; ‡ Department of Emergency Medicine, Shenzhen University General Hospital, Shenzhen University, Shenzhen 518051, China; § AIM Honesty Biopharmaceutical Co., Ltd, Dalian 116620, China; ∥ Department of Infectious Diseases, Imperial College London, London W12 0NN, U.K.

**Keywords:** HBV vaccine, virus-like particle, adjuvant, cationic polymer, polyphosphoester, immune response

## Abstract

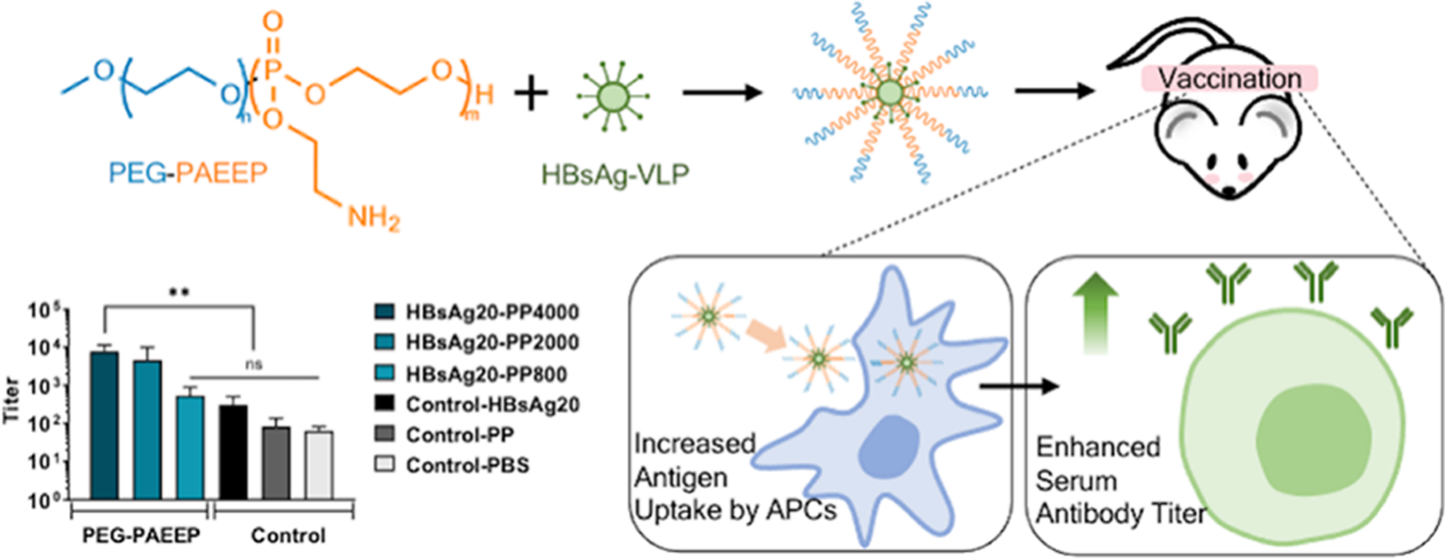

Virus-like particle (VLP)-based vaccines are required to be associated with a suitable adjuvant to potentiate their immune responses. Herein, we report a novel, biodegradable, and biocompatible polyphosphoester-based amphiphilic cationic polymer, poly(ethylene glycol)-*b*-poly(aminoethyl ethylene phosphate) (PEG–PAEEP), as a Hepatitis B surface antigen (HBsAg)-VLP vaccine adjuvant. The polymer adjuvant effectively bound with HBsAg-VLP through electrostatic interactions to form a stable vaccine nanoformulation with a net positive surface charge. The nanoformulations exhibited enhanced cellular uptake by macrophages. HBsAg-VLP/PEG–PAEEP induced a significantly higher HBsAg-specific IgG titer in mice than HBsAg-VLP alone after second immunization, indicative of the antigen-dose sparing advantage of PEG–PAEEP. Furthermore, the nanoformulations exhibited a favorable biocompatibility and *in vivo* tolerability. This work presents the PEG–PAEEP copolymer as a promising vaccine adjuvant and as a potentially effective alternative to aluminum adjuvants.

## 1 Introduction

Vaccination has been one of the most effective approaches to combating the spread of infectious diseases, thus preventing hospitalization and fatality.^1,2^ Although the majority of the marketed vaccines are based on attenuated and inactivated whole pathogens, there is an increasing interest in the development of vaccines based on specific antigenic components from pathogens, known as microbial subunits. Given that such vaccines do not contain any “live” pathogen components, they have no risk of introducing a disease. Hence, it provides a safer and more stable alternative to whole pathogen vaccines.^3^ Among subunit antigens, virus-like particles (VLPs), formed by self-assembled viral proteins, have attracted particular interest due to their superior immunogenicity.^4,5^ The Hepatitis B surface antigen (HBsAg) and the human papillomavirus (HPV) vaccines are currently the most notable examples of VLP-based vaccines.^6−8^ However, despite their high immunogenicity, VLP-based vaccines are necessarily associated with a suitable adjuvant to potentiate the immune responses.^9^

Adjuvants have been reported to increase vaccine immunogenicity, prolong the duration of prophylaxis, and allow for the reduced amount of antigens required (antigen-dose sparing), thereby reducing the requirement for boost vaccinations.^10^ Adjuvants can function as a reservoir for antigens and prolong the duration at which antigens are presented to immune cells. Immune responses can thus be increased before antigens are cleared from the body. In addition, as immune modulators, adjuvants can facilitate inflammatory reactions at the delivery site, with primarily localized and transient effects to promote the recruitment and activation of immune cells.^11^

To date, most of the licensed VLP-based vaccines utilize classic aluminum adjuvants as they are effective at enhancing antibody responses through complex cellular interactions and local antigen depot effects through electrostatic adsorption.^12^ The adsorbed antigens can better maintain their physiochemical properties, improving the stability of vaccine formulations.^13^ In addition, upon injection, antigen particles bound with adjuvants can diffuse at a much slower rate from the site of administration, allowing antigen-presenting cells (APCs) to accumulate for better antigen recognition, endocytic processing, and presentation. Global coverage of the 3-dose Hepatitis B vaccine in 2019 was reported to reach 85%.^14^ Aluminum adjuvants are commonly used in Hepatitis B vaccines worldwide. Five Hepatitis B vaccines (PreHevbrio, Recombivax HB, Engerix B, Twinrix, and Heplisav-B) have been approved for use in the U.S.,^15^ and all but Heplisav-B utilize aluminum adjuvants.^16−20^ All three licensed Hepatitis B vaccines (Fendrix, Engerix B, and HBvaxPRO) in the U.K. incorporate aluminum adjuvants.^21^ Additionally, the Hepatitis B vaccines used in China, produced from *Saccharomyces cerevisiae*, Hansenula polymorpha, and Chinese hamster ovary cells, are all adjuvated with aluminum hydroxide.^22,23^ However, aluminum adjuvants are prone to produce adverse reactions near the injection site and on a systemic level.^12^ The localized side effects include inflammation, leading to pain and tenderness.^24^ On a systemic level, aluminum adjuvants have drawbacks including possible allergic reactions, immune bias,^12^ and, in some cases, inability to enhance immune responses on a cellular level.^25^ There is also an additional concern about the formation of long-term tissue depots,^12^ leading to chronic toxicity associated with the accumulation of aluminum in the human body that may cause neurodegeneration and renal dysfunction.^26,27^ Furthermore, certain challenging antigens and weak immune responders may require further optimization of the adjuvants to maximize the strength and duration of protective immune responses.

In recent decades, a considerable amount of research effort has been put into developing alternative vaccine adjuvants. A variety of delivery systems, such as liposomes and emulsions, have been applied as vaccine adjuvants.^28,29^ For example, adjuvants AS03 and MF59 from GSK are based on oil-in-water (O/W) emulsions. However, they are usually assembled from multiple components, and overall quality control is more complicated. Additionally, they have the potential to cause local toxicities including severe injection-site pain due to local tissue damage followed by severe inflammatory reactions, which, in some cases, could produce a sterile granuloma or ulceration at the injection site.^30^ Some general systemic inflammation symptoms, including fever, headache, and malaise, could also occur.^31^ Recently, polymeric nanoparticles prepared from biodegradable polymers have been extensively researched in the vaccine field. Their adjuvant efficacy can be manipulated by the polymer structure, amphiphilicity, and surface charge of self-assembled nanostructures. Widely recognized as vaccine delivery systems, polymeric adjuvants are also emerging immunostimulators capable of enhancing immune responses. Their interactions with APCs are attributed to their chemical compositions and particulate nature.^32^ For example, chitosan has been reported as a vaccine adjuvant and its degree of deacetylation, molecular weight, and level of impurities and contaminants could influence its adjuvant efficacy and even hinder its clinical use.^33^ All in all, it is necessary to develop an efficient polymeric adjuvant with controllable properties as well as minimal side effects for VLP-based vaccines.^34^

Biodegradable polyphosphoesters (PPEs) have been widely investigated for applications in drug delivery and tissue engineering for several decades. The U.S. FDA-approved synthetic polyesters have favorable biocompatibility and degradability.^35,36^ Their versatile structures can be easily controlled by functional modifications of monomers.^36,37^ PPE-based nanodelivery systems have been previously demonstrated to display enhanced cellular uptake and controlled drug release in tumor therapy.^38,39^

**Figure 1:**
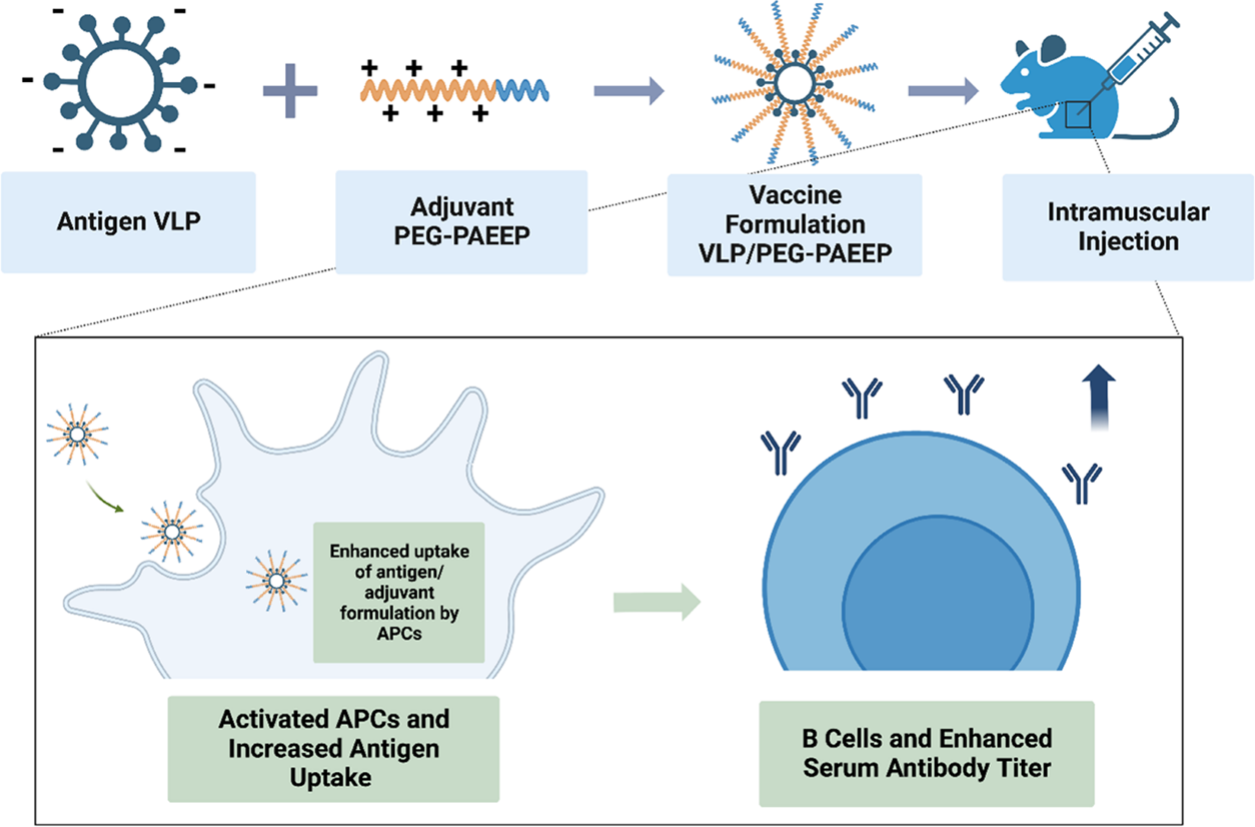
Schematic illustration of the VLP/PEG–PAEEP vaccine formulation and its immunostimulatory effect. Created with BioRender.com.

Herein, we report a novel PPE-based polymer that can serve as a vaccine delivery system with immunostimulatory adjuvant properties (Figure 1). The diblock copolymer, consisting of a poly(ethylene glycol) (PEG) block and a poly(aminoethyl ethylene phosphate) (PAEEP) block bearing pendant ethanolamine groups, was synthesized by ring-opening polymerization. The PEG–PAEEP is a cationic polymer with a readily controllable molecular weight and structure. This positively charged biodegradable polymer was mixed with the negatively charged antigen HBsAg-VLP to form nanosize particles. The cellular uptake of the HBsAg-VLP/PEG–PAEEP particles by macrophages was exhibited by laser scanning confocal microscopy. Furthermore, the immunostimulatory properties of various HBsAg-VLP/PEG–PAEEP nanoformulations were investigated in mice, and their adjuvant capacity was determined via quantification of the anti-HBsAg antibodies produced. Their biocompatibility and *in vivo* tolerability profiles were evaluated by hemolysis and cytotoxicity assays as well as mouse body weight measurements during immunization.

## 2 Materials and Methods

### 2.1 Materials

2-Chloro-2-oxo-1,3,2-dioxaphospholane (COP) was purchased from Tokyo Chemical Industry Co., Ltd. (Tokyo, Japan). 1,5,7-Triazabicyclo[4.4.0]dec-5-ene (TBD), *N*-Boc-ethanolamine, fluorescein isothiocyanate (FITC), mPEG_5k_–OH, paraformaldehyde solution (4%, w/v), bovine serum albumin (BSA), triethylamine (TEA), diethyl ether, and trifluoroacetic acid (TFA) were purchased from Sigma-Aldrich (Dorset, U.K.). Trypsin-EDTA (0.25%, w/v), fetal bovine serum (FBS), and penicillin-streptomycin (100×) were bought from Gibco (Carlsbad, CA). Dulbecco’s phosphate-buffered saline (D-PBS) and Dulbecco’s modified Eagle’s medium (DMEM) were obtained from HyClone Lab (Logan, UT). 3,3′,5,5′-Tetramethylbenzidine was obtained from Thermo Fisher Scientific (MA). Phosphate-buffered saline (PBS) was purchased from Shanghai Titan Technology Co., Ltd. (Shanghai, China). The RAW264.7 mouse macrophage cell line was purchased from ATCC (Manassas, VA). Defibrinated sheep red blood cells (RBCs) were purchased from TCS Biosciences (Buckinghamshire, U.K.). The HBsAg-VLP vaccine was gifted by AIM Honesty Biopharmaceutical Co., Ltd. (AIM, Dalian, China). The Imject Alum Adjuvant was obtained from Thermo Fisher (Rockford, IL). CD11c Microbeads Ultrapure (mouse), MidiMACS Starting Kit (LS), MACS BSA Stock Solution, and autoMACS Rinsing Solution were purchased from Miltenyi Biotec Technology & Trading (Shanghai) Co., Ltd. The mouse spleen lymphocyte separator kit was obtained from Solarbio (Beijing, China). 2-(4-Amidinophenyl)-6-indolecarbamidine dihydrochloride (DAPI) was purchased from GuangZhou Sopo Biological Technology Co., Ltd. (China).

### 2.2 Cell Culture

Mouse macrophage RAW264.7 cells were grown in DMEM supplemented with 10% (v/v) FBS and 100 U mL^–1^ Penicillin/Streptomycin unless specified otherwise. The RAW264.7 cells were trypsinized using trypsin–EDTA and maintained in a humidified incubator with 5% CO_2_ at 37 °C.

### 2.3 Polymer Synthesis and Characterization

PEG–PAEEP was synthesized by ring-opening polymerization according to the previously reported method.^38,39^ First, COP, *N*-Boc-ethanolamine, and TEA were mixed at a molar ratio of 1:1:2 and kept stirring under the N_2_ atmosphere at 0 °C for 24 h. After filtration and washing with diethyl ether several times, the monomer 2-(*N*-Boc-ethanolamine)-2-oxo-1,3,2-dioxaphospholane (*N*-Boc-EAOP) was obtained. Then, mPEG_5k_–OH and *N*-Boc-EAOP were reacted at 50 °C for 1 h to obtain PEG-*N*-Boc-PAEEP using TBD as a catalyst. The Boc protecting group was removed by dissolving PEG-*N*-Boc-PAEEP in TFA at room temperature for 3 h. Finally, the product was purified by precipitation in diethyl ether for 3 times. The PEG–PAEEP polymer structure was confirmed by proton nuclear magnetic resonance (^1^H NMR) spectroscopy, and the degree of polymerization of the PAEEP block was calculated. The polymer was also analyzed by Fourier transform infrared (FTIR) spectroscopy.

The biocompatibility of the PEG–PAEEP polymer was investigated by a hemolysis assay. Briefly, defibrinated sheep RBCs were washed with PBS (306 mOsm L^–1^, pH 7.4) 3 times. The cell pellets were resuspended into a suspension with PBS (306 mOsm L^–1^, pH 7.4) with a final RBC concentration of 1–2 × 10^8^ RBCs per mL.^40^ PEG–PAEEP was added to the RBC suspension to reach the specified final polymer concentrations from 1 to 20 mg mL^–1^. A separate sample was prepared for comparison by adding the Imject Alum Adjuvant into the RBC suspension at a concentration of 500 μg mL^–1^ used for *in vivo* application (containing an equivalent amount of positive charges compared with the PEG–PAEEP polymer at 4 mg mL^–1^). After incubation in a shaking water bath at 37 °C for 1 h, the samples were centrifuged at 3500 rpm for 3 min, and the supernatant was then transferred to a cuvette. Treatment of the RBC suspension with deionized water was used as the positive control and treatment with PBS (306 mOsm L^–1^, pH 7.4) as the negative control. The absorbance (*A*) of the supernatant was measured at 540 nm by using a UV–vis spectrophotometer (GENESYS 10S UV–vis, Thermo Scientific). The relative hemolysis was calculated according to the following equation:
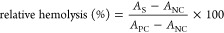
where *A*_S_, *A*_NC_, and *A*_PC_ represent the absorbance of the sample, the negative control, and the positive control, respectively.

The cytotoxicity of PEG–PAEEP at different concentrations toward the mouse macrophage cell line RAW264.7 was evaluated using a CellTiter-Glo assay. A 200 μL amount of RAW264.7 cells were seeded in a 96-well plate (Corning) at a density of 1.0 × 10^4^ cells per well. After culture for 24 h, the cells were treated for 4 h with the filter-sterilized PEG–PAEEP solution at specific concentrations in serum-free DMEM medium. The cell cytotoxicity of the aluminum adjuvant sample containing an equivalent amount of positive charges to the PEG–PAEEP polymer sample was also tested for comparison. The cells were then washed with D-PBS 3 times and replenished with 100 μL of fresh complete DMEM per well, and further incubated overnight. The cells were then equilibrated at room temperature for 30 min before the addition of the CellTiter-Glo reagent (100 μL per well). The plate was then put on to a shaker, and its content was mixed after shaking for 2 min to ensure complete cell lysis. The content was then transferred to an opaque-walled white 96-well plate and equilibrated for 10 min at room temperature to stabilize the luminescent signal before the luminescence of each well was measured using a spectrofluorometer (GloMax-Multi Detection System, Promega). The readings of the wells with complete DMEM but no cells were measured as the background luminescence. Values of the viability of the polymer-treated cells were expressed as a percentage relative to the control cells treated with DMEM only.

### 2.4 Preparation and Characterization of HBsAg-VLP/PEG–PAEEP Vaccine Formulations

HBsAg-VLP/PEG–PAEEP vaccine formulations were designed and grouped according to different antigen concentrations, adjuvant concentrations, and antigen-to-adjuvant mass ratios used. Various vaccine formulations were prepared by mixing different mass ratios of HBsAg-VLP and PEG–PAEEP in D-PBS, as summarized in Table 1. For example, the HBsAg20-PP4000 formulation was prepared at the final concentrations of HBsAg-VLP and PEG–PAEEP at 20 and 4000 μg mL^–1^, respectively. The other vaccine formulations were prepared according to the same naming rule. The particle size and polydispersity (PDI) of HBsAg-VLP and HBsAg-VLP/PEG–PAEEP were analyzed by a Zetasizer μV light scattering detector (Malvern, U.K.), and their ζ-potentials were measured by a ZetaPALS analyzer (Brookhaven). The morphologies of HBsAg-VLP and HBsAg-VLP/PEG–PAEEP were observed by transmission electron microscope (TEM) using a JEOL STEM/TEM 2100Plus instrument

**Table 1:** Composition Types and Concentrations of Different Vaccine Formulations

formulation	HBsAg (μg mL^–1^)	PEG–PAEEP (PP, μg mL^–1^)	aluminum (Al, μg mL^–1^)
HBsAg20-PP4000	20	4000	
HBsAg10-PP4000	10	4000	
HBsAg5-PP4000	5	4000	
HBsAg20-PP2000	20	2000	
HBsAg10-PP2000	10	2000	
HBsAg5-PP2000	5	2000	
HBsAg20-PP800	20	800	
HBsAg10-PP1000	10	1000	
HBsAg5-PP500	5	500	
HBsAg20-Al500	20		500
control-HBsAg20	20		
control-PP		4000	
control-Al			500
control-PBS			

### 2.5 Uptake of HBsAg-VLP/PEG–PAEEP Vaccine Formulations by APCs

HBsAg-VLP was conjugated with a fluorescent dye, FITC (mass ratio of HBsAg-VLP to FITC at 10), to evaluate the cellular uptake of HBsAg-VLP and HBsAg-VLP/PEG–PAEEP. Briefly, 1 mg of HBsAg-VLP was dissolved in 1 mL of PBS buffer (0.01 M, pH 8.0), to which was added dropwise 0.1 mL of FITC-containing DMSO at 1.0 mg mL^–1^. The reaction mixture was kept stirring at room temperature for 24 h in the dark. DMSO and free FITC were removed by dialysis (MWCO = 3 kDa) against deionized water to obtain the FITC-labeled HBsAg-VLP.

Mouse splenocytes were extracted, and dendritic cells (DCs) were isolated using magnetic bead sorting. Mice were euthanized, and the spleen was aseptically excised and transferred to a Petri dish with the chilled MACS BSA stock solution consisting of PBS and 10% (v/v) BSA. The spleen tissue was pressed through a strainer using a syringe barrel to obtain a single-cell suspension. After centrifugation with the mouse spleen lymphocyte separator kit according to the manufacturer’s protocol, the spleen lymphocytes were obtained and resuspended in a MACS BSA stock solution. Magnetic beads conjugated with CD11c antibodies were added to the cell suspension and incubated for 15 min. The cell-bead mixture was then placed on a magnetic separation device, allowing the magnetically labeled DCs to adhere while the unbound cells were removed. The bead-bound DCs were washed and eluted from the magnetic beads using the autoMACS rinsing solution. The eluted DCs were then collected, washed again, and prepared for further analysis. Briefly, 3 × 10^5^ DCs collected from the mouse spleen were seeded in a 6-well plate and incubated in completed DMEM for 24 h. After the spent culture medium was replaced with fresh serum-free DMEM medium, the HBsAg, HBsAg20-PP800, and HBsAg20-PP4000 formulations were added at an equivalent HBsAg concentration of 1 μg per well, respectively. The DCs were incubated for 2 h and then washed three times with PBS. Flow cytometry was performed to assess the percentage of FITC-positive DCs using a flow cytometer (Sparrow, China). The samples were excited at 490 nm, and the emission was collected in the 520–530 nm band.

A 2 mL amount of RAW264.7 cells was seeded in a 35 mm glass bottom plate (MatTek) at a density of 3 × 10^5^ per dish. After culturing for 24 h, the spent medium was removed and replaced with 2 mL of 0.22 μm filter-sterilized serum-free DMEM containing FITC-labeled HBsAg-VLP or HBsAg20-PP4000 at an equivalent HBsAg-VLP concentration of 1 μg mL^–1^. After incubation for 4 h, the cells were washed with D-PBS 3 times and then stained with a DAPI solution at a concentration of 5 μg mL^–1^ for 10 min. The cells were rinsed with D-PBS 3 times before imaging under a laser scanning confocal microscope (Leica SP8 Inverted). FITC was excited using a 490 nm laser, and the emission at 525 nm was collected. DAPI was excited using a 340 nm laser, and the emission at 488 nm was collected. The intracellular FITC mean fluorescence intensity (MFI) was analyzed by ImageJ software.

### 2.6 Immunization Studies

Female BALB/c mice (8 weeks old) were purchased from Liaoning Changsheng Biotechnology Co., Ltd. (China). The animals were provided with food and water, and all animal experiments were approved by the Committee on the Ethics of Animal Experiments of the AIM (AIM Honesty Biopharmaceutical Co., Ltd.) and conducted in compliance with the recommendations in the Guide for the Care and Use of Laboratory Animals of the AIM Ethics Committee. Groups of eight mice were used to test different HBsAg-VLP formulations. Briefly, mice were first intramuscularly (IM) injected at their right back leg with different HBsAg-VLP/PEG–PAEEP formulations (Table 1) at the fixed injection volume of 100 μL, and the boost was then carried out at day 14. At 2 weeks post the boost, anti-HBsAg IgG antibodies in serum were measured by the enzyme-linked immunosorbent assay (ELISA). The mouse body weights were also monitored during immunization.

### 2.7 Measurement of HBsAg-Specific Antibodies and Cytokine by ELISA

During immunization of female BALB/c mice, blood was harvested from the tail vein on day 28 and centrifuged to obtain serum. HBsAg-specific IgG antibodies were then measured by ELISA. In brief, each well of a 96-well flat-bottom plate was coated using 100 μL of carbonate buffer (50 mM Na_2_CO_3_–NaHCO_3_) containing 5 μg mL^–1^ HBsAg-VLP at 4 °C for 15 h. Each well of the plate was then washed five times with PBST (*i.e*., PBS containing 0.05% (w/w) Tween 20) and blocked with 200 μL of 1% (w/v) BSA at 37 °C for 90 min. Serum was diluted 2-fold with PBS, and 100 μL of the diluted serum was added to each well. After incubation at 37 °C for 45 min, each well was washed five times with PBST. 100 μL portion of peroxidase-conjugated Goat anti-Mouse IgG antibodies was added in each well at a dilution fold of 1:7500 and incubated at 37 °C for 30 min. Unbound antibodies were washed away with PBST, and each well was then filled with 200 μL of 3,3′,5,5′-tetramethylbenzidine solution followed by incubation at 37 °C in the dark for 15 min. After 50 μL of 2 M H_2_SO_4_ was added to each well to stop the enzymatic reaction, the optical density (OD450) values were measured by a SpectraMax M3 microplate reader (Molecular Devices) with a 450 nm filter. If the OD450 value of a diluted serum sample was less than 2.1-fold that of the negative control serum or below 0.105, then the dilution ratio of the sample was taken as the final titer.

Serum concentrations of interferon-γ (IFN-γ) in the female BALB/c mice after the boost immunization intramuscularly with different vaccine formulations at the fixed injection volume of 100 μL were also measured by the ELISA kit according to the manufacturer’s protocol.

### 2.8 Statistical Analysis

The quantitative data were repeated in triplicate (*n* = 3) and expressed as mean values ± standard deviations encompassing a 95% confidence interval. Statistical analysis was performed using one-way analysis of variance (ANOVA) with a Tukey’s post hoc test and Student’s *t* test to evaluate the statistical significance of difference, with a value of *p* < 0.05 considered to be statistically significant. Analysis of quantitative data was performed in Graph-Pad Prism 8 and Microsoft Excel. The presented TEM and confocal microscopy images were representative in the given experimental samples.

## 3 Results and Discussion

### 3.1 Polymer Synthesis and Characterization

**Figure 2:**
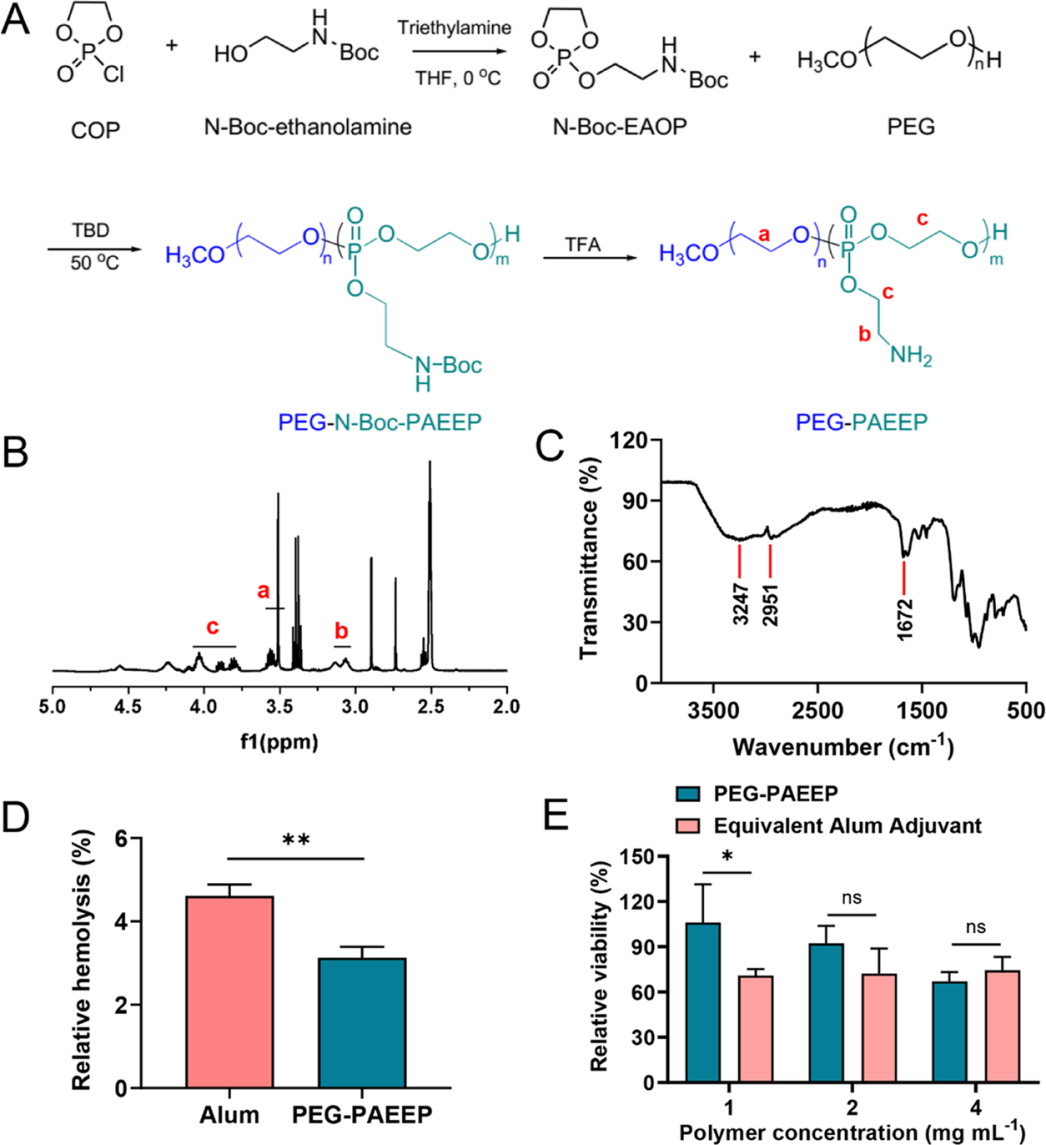
(A) Synthesis of the PEG–PAEEP polymer by ring-opening polymerization. (B) ^1^H NMR spectrum and (C) FTIR spectrum of PEG–PAEEP. (D) Relative hemolysis of RBCs incubated with PEG–PAEEP at 4 mg mL^–1^ for 1 h and (E) relative viabilities of RAW264.7 cells incubated with PEG–PAEEP at various concentrations for 4 h, as compared with the aluminum adjuvant containing an equivalent amount of positive charges. Statistical analysis was performed using the Student’s *t* test and one-way ANOVA test. *p < 0.05, **p < 0.01, and ns represents no significant difference between two groups, respectively.

A positively charged biodegradable diblock copolymer, PEG–PAEEP, was designed as an effective vaccine adjuvant to improve the immune responses of antigens through protecting them from degradation and enhancing their cellular uptake by APCs. The PEG–PAEEP was synthesized by ring-opening polymerization of the monomer, *N*-Boc-EAOP, using TBD as a catalyst (Figure 2A). mPEG_5k_–OH with one of the most widely used molecular weights in drug delivery was chosen as an initiator. The ^1^H NMR spectrum of the obtained PEG–PAEEP polymer is depicted in Figure 2B. The signals of methylene protons (−OC***H***_2_C***H***_2_, a) on the PEG block and methylene protons (−C***H***_2_NH_2_, b) on the PAEEP block were observed at 3.52 and 3.10–3.20 ppm, respectively. The resonances at 3.75–4.20 ppm represented the peaks corresponding to methylene protons (−POC***H***_2_C***H***_2_O–, c) on the phosphoester backbone and methylene protons (−P–OC***H***_2_CH_2_NH_2_, c) on the side chains. Based on the integrals of proton resonance of methylene groups (−OC***H***_2_C***H***_2_, a) on the PEG block and methylene groups (−C***H***_2_NH_2_, b) on the PAEEP block, the molecular weight of the PAEEP block was calculated to be 26.5 kDa. The FTIR spectrum of the PEG–PAEEP polymer (Figure 2C) shows the −NH_2_ stretching absorption bands at 3247 and 2951 cm^–1^, while the −NH_2_ bending absorption band is at 1672 cm^–1^.

The biocompatibility of the PEG–PAEEP polymer was evaluated by using a hemolysis assay. As shown in Figure 2D, PEG–PAEEP at 4 mg mL^–1^ displayed negligible hemolysis after 1 h of incubation, which was significantly lower (*p* < 0.01) than the aluminum adjuvant containing an equivalent amount of positive charges. The hemolytic activity of PEG–PAEEP increased only marginally, remaining at a very low level of below 5%, with an increase in the polymer concentration to as high as 20 mg mL^–1^ (Figure S1A). No significant increase in hemolysis was observed when the duration for the incubation of RBCs with PEG–PAEEP was increased from 1 to 5 h (Figure S1B). Furthermore, the cytotoxicity of PEG–PAEEP toward RAW264.7 cells was measured using a CellTiter-Glo assay. Figure 2E shows that, as compared with the aluminum adjuvant containing the equivalent amount of positive charges, PEG–PAEEP exhibited significantly higher cell viability (*p* < 0.05) at a polymer concentration of 1 mg mL^–1^, while no significant difference was observed at the higher polymer concentrations.

### 3.2 Preparation and Characterization of HBsAg-VLP/PEG–PAEEP Vaccine Formulations

**Figure 3:**
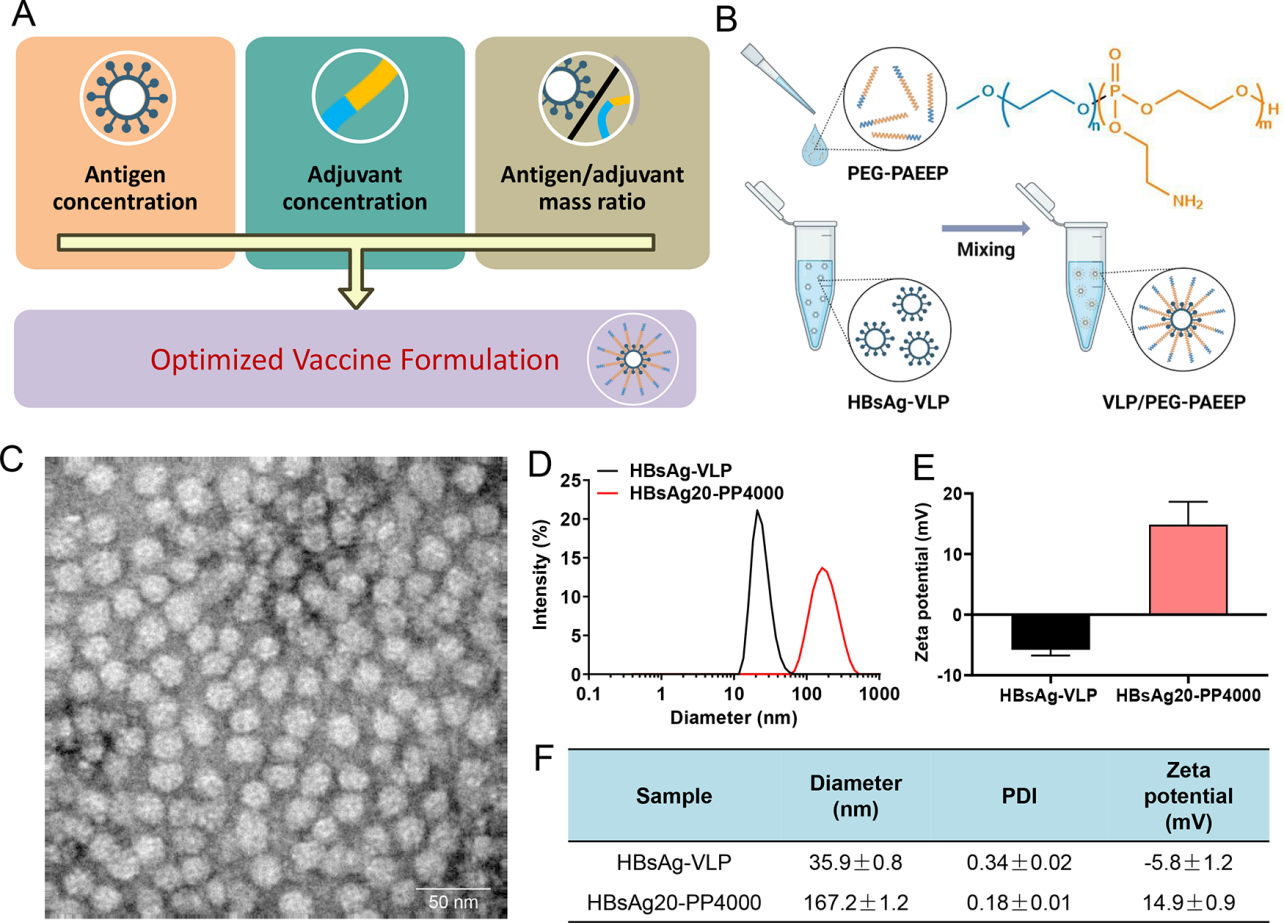
(A) Schematic graph of the factors influencing the grouping for vaccine formulations. (B) Schematic graph of the preparation of the HBsAg/PEG–PAEEP vaccine formulations. Created with Biorender.com. (C) TEM micrograph of HBsAg-VLP. (D–F) Hydrodynamic diameter distributions, PDIs, and ζ-potentials of HBsAg-VLP and HBsAg20-PP4000 formulations.

**Figure 4:**
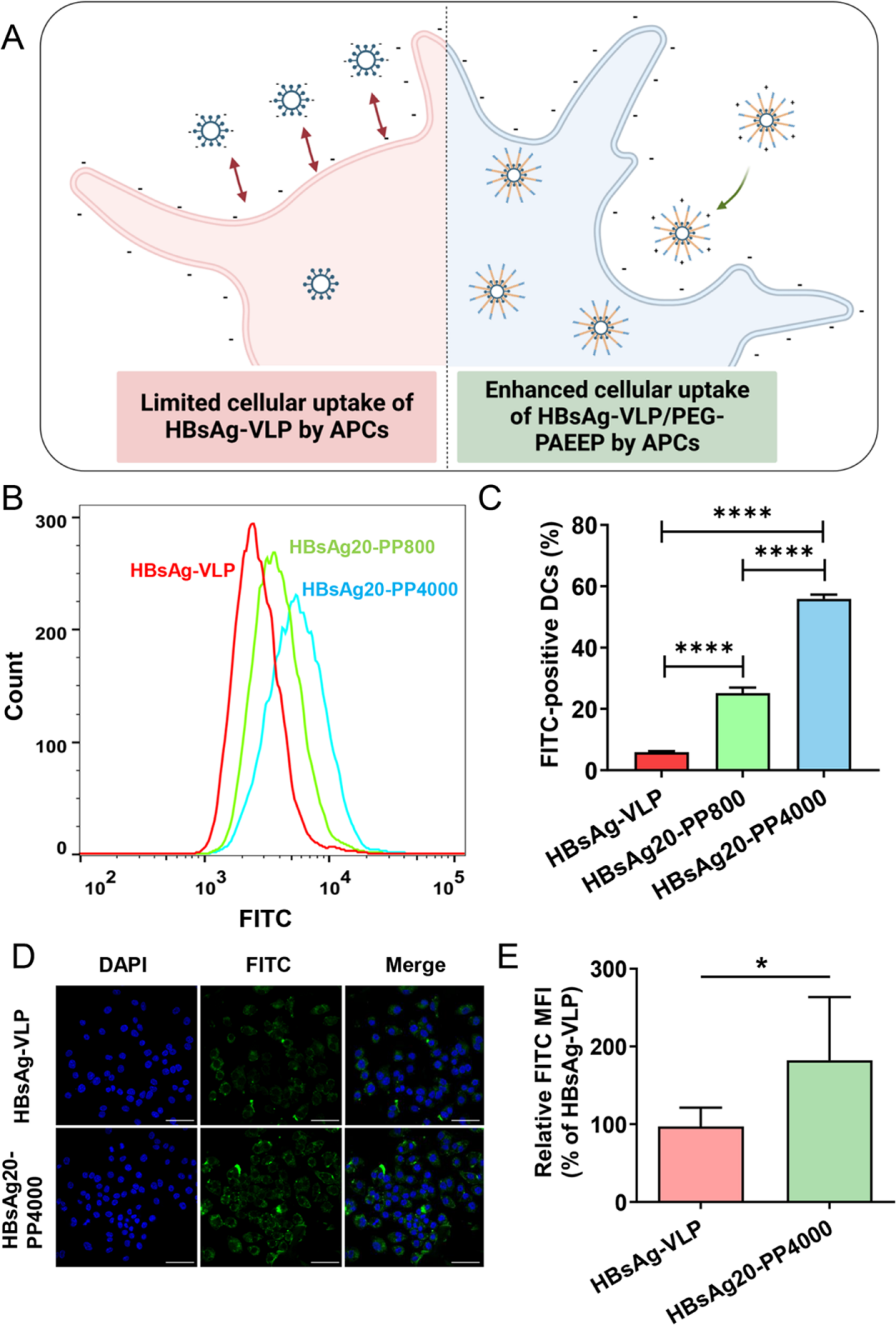
(A) Schematic graph of the enhanced cellular uptake of the HBsAg-VLP/PEG–PAEEP vaccine formulation by APC cells compared with HBsAg-VLP. Created with Biorender.com. (B) Flow cytometry histogram profiles and (C) percentages of FITC-positive DCs after 2 h of incubation with FITC-labeled HBsAg-VLP, HBsAg20-PP800, and HBsAg20-PP4000 at an equivalent HBsAg-VLP concentration of 1 μg mL^–1^. (D) Confocal microscopy images (scale bar, 40 μm) and (E) the relative intracellular FITC mean fluorescence intensity (MFI) as analyzed with the use of ImageJ, of RAW264.7 cells incubated with FITC-labeled HBsAg-VLP or HBsAg20-PP4000 nanoparticles at the equivalent HBsAg-VLP concentration of 1 μg mL^–1^ for 4 h. Statistical analysis was performed using the Student’s *t* test and one-way ANOVA test. *p < 0.05 and ****p < 0.0001, respectively.

**Figure 5:**
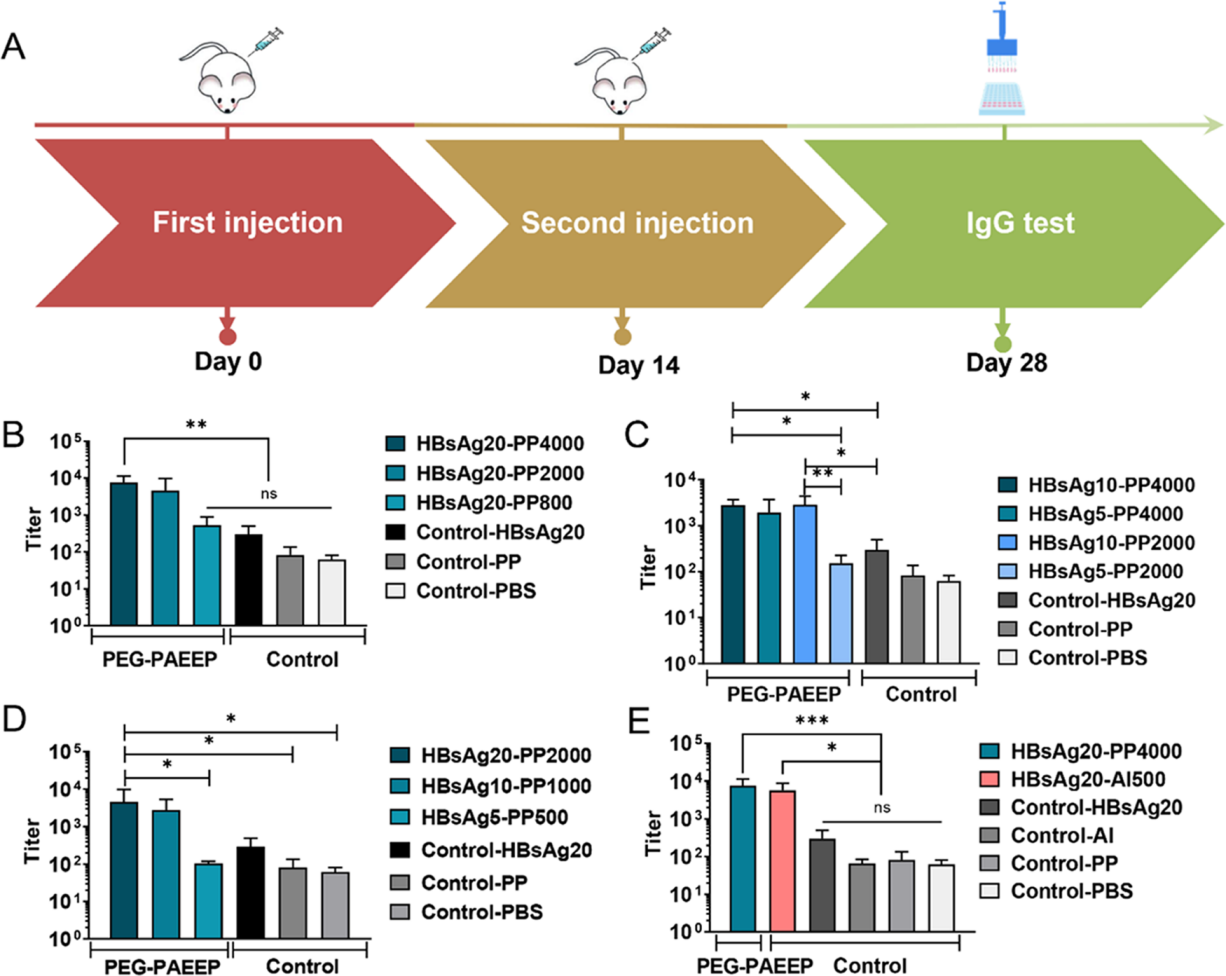
(A) Schematic illustration of the immunogenicity experiments in female BALB/c mice immunized with IM with various vaccine formulations at the fixed injection volume of 100 μL. (B) Serum anti-HBsAg IgG titers of the groups that were prepared with the fixed HBsAg concentration (20 μg mL^–1^), but different PEG–PAEEP concentrations at day 28. (C) Serum anti-HBsAg IgG titers of the groups that were prepared with fixed PEG–PAEEP concentrations but different HBsAg concentrations at day 28. (D) Serum anti-HBsAg IgG titers of the groups that were prepared at the fixed HBsAg to PEG–PAEEP mass ratio on day 28. (E) Serum anti-HBsAg IgG titers of the HBsAg20-Al500 and HBsAg20-PAEEP4000 groups on day 28. Statistical analysis was performed using the one-way ANOVA test. *p < 0.05, **p < 0.01, ***p < 0.001, and ns represents no significant difference between two groups, respectively.

To improve the immunogenicity of vaccine formulations, the polymeric adjuvant should be designed to form a nanoparticle with antigens as an “antigen tank”, which serves two main purposes: to offer protection of antigens from degradation and to facilitate their cellular uptake by APCs. As shown in Figure 3A, different HBsAg-VLP/PEG–PAEEP formulations were grouped by controlling the antigen concentration, polymer concentration, and HBsAg-VLP to PEG–PAEEP mass ratio to investigate the effects of vaccine compositions on potentiation of immune responses. The vaccine formulations were prepared by the formation of nanoparticles by mixing HBsAg-VLP and PEG–PAEEP at different mass ratios (Figure 3B). The specific composition types and concentrations of different vaccine formulations are summarized in Table 1. Figure 3C shows the spherical morphology of the HBsAg-VLP particles. The TEM size of HBsAg-VLP (Figure 3C) was consistent with its dynamic light scattering (DLS) diameter of 35.9 ± 0.8 nm (Figure 3D,3F). Figures 3 and 4 show the characterization data of the representative formulation, HBsAg20-PP4000 (with 20 μg mL^–1^ of HBsAg-VLP and 4000 μg mL^–1^ of PEG–PAEEP), which exhibited the optimal immune response among all of the vaccine formulations tested in the following *in vivo* immunization experiments as shown in Figure 5.

As a comparison in Figure 3D,F, after the formulation of HBsAg-VLP with PEG–PAEEP as a polymeric adjuvant, the diameter of the obtained HBsAg20-PP4000 increased to 167.2 ± 1.2 nm, indicative of the successful formation of vaccine nanoformulations. The lower PDI of HBsAg20-PP4000 at 0.18 ± 0.01 suggests the formation of monodispersed nanoparticles. Figure 3E,3F shows that the HBsAg-VLP antigen displayed a ζ-potential of −5.8 ± 1.2 mV, while its formulation with the positively charged PEG–PAEEP led to an increase of ζ-potential to 14.9 ± 0.9 mV, further confirming the successful vaccine nanoformulation. This positive surface charge could be beneficial in promoting interactions of the obtained nanoformulations with the negatively charged cell membrane, thus improving the cellular uptake by APCs. Furthermore, degradation of active vaccine ingredients can cause a loss or decrease in antigen efficacy. Herein, the combination of negatively charged HBsAg-VLP antigen with the positively charged PAEEP block of the PEG–PAEEP copolymer resulted in the formation of stable nanoparticles, as presented in Figure 3. Other researchers have reported that encapsulation of susceptible proteins in the nanoparticle core could reduce the accessibility of protein payload during delivery, thereby minimizing enzymatic degradation.^41^ It has also been extensively reported that PEGylation can enhance colloidal and protein stability and prolong blood circulation through producing the hydration layer and steric shielding effect on the nanoparticle surface.^38,39,42,43^

### 3.3 Uptake of HBsAg-VLP/PEG–PAEEP Vaccine Formulations by APCs

FITC-labeled HBsAg-VLP was used to evaluate the cellular uptake of the vaccine formulations with and without PEG–PAEEP as a polymeric adjuvant. First, DCs were used as typical APCs to evaluate the uptake of HBsAg-VLP, HBsAg20-PP800, and HBsAg20-PP4000 at an equivalent antigen concentration of 1 μg mL^–1^ by flow cytometry. Figure 4B,C shows that, after incubation with HBsAg20-PP4000 for 2 h, the percentage of FITC-positive DCs reached 55.9 ± 1.4%, which was 2.23 and 9.47 times higher than the HBsAg20-PP800 (25.1 ± 1.8%, *p* < 0.0001) and HBsAg-VLP (5.9 ± 0.4%, *p* < 0.0001) groups, respectively. This suggests that the cationic PEG–PAEEP polymer can facilitate the antigen intracellular uptake as an adjuvant, with a higher polymer concentration at 4000 μg mL^–1^ leading to significantly higher antigen uptake as compared with the lower polymer concentration at 800 μg mL^–1^. The improved cellular uptake of antigen by APCs should be attributed to the net positive surface charge of the nanoformulation,^44^ as presented in Figures 3 and 4A.

Then, macrophage RAW264.7 cells were imaged by laser scanning confocal microscopy after 4 h of incubation with HBsAg-VLP and HBsAg20-PP4000, respectively, to further validate the polymeric adjuvant-induced antigen uptake by APCs. As presented in Figure 4D, the RAW264.7 cells treated with HBsAg20-PP4000 displayed a stronger FITC green fluorescence intensity than those treated with HBsAg-VLP alone. The DAPI nuclear staining was employed to demonstrate the antigen localization in the cytoplasm. Quantitative analysis of the intracellular FITC mean fluorescence intensity (MFI) in the confocal microscopy images confirmed that the macrophage cellular uptake of HBsAg20-PP4000 was significantly higher than HBsAg-VLP (*p* < 0.05), as shown in Figure 4E. The improved cellular uptake of antigen by APCs has prompted us to do the following *in vivo* immunization for evaluation of the potency of PEG–PAEEP as the adjuvant for HBsAg-VLP.

### 3.4 Immunization Studies

Female BALB/c mice were immunized according to the timeline illustrated in Figure 5A using various vaccine formulations that were grouped by the antigen concentration, polymer concentration, and polymer-to-antigen mass ratio used, as listed in Table 1. At 2 weeks post the boost (*i.e*., day 28), the serum anti-HBsAg IgG of these groups were quantified by ELISA. First, the potency of PEG–PAEEP as the adjuvant was evaluated when the fixed HBsAg-VLP concentration of 20 μg mL^–1^ was used to prepare the vaccine formulations (Figure 5B). When a low PEG–PAEEP concentration of 800 μg mL^–1^ (HBsAg20-PP800) was used, the resulting anti-HBsAg IgG titer was comparable to that caused by HBsAg-VLP alone. Further increasing PEG–PAEEP concentration to 4000 μg mL^–1^ (HBsAg20-PP4000) led to a significantly increased anti-HBsAg IgG titer (*p* < 0.01), which was attributed to the adjuvant-enhanced uptake by APCs as presented in Figure 4. This suggests that the cationic PEG–PAEEP polymer can effectively improve the immune responses of HBsAg-VLP and that at a fixed HBsAg-VLP concentration, the serum anti-HBsAg IgG titer increases with the polymer concentration until the efficacy of the given amount of antigen is maximized. The finding is in good agreement with the report by other researchers that the positive surface charge of a nanoparticle adjuvant ASP–PLGA-PEI, a polyethylenimine (PEI)-coated poly(lactic-*co*-glycolic acid) (PLGA) nanoparticle with the encapsulated immunostimulant Angelica sinensis polysaccharide (ASP), can facilitate the uptake by APCs and thus improve immune responses of H9N2 vaccine in chickens.^44^

Figure 5C shows the potency of the polymeric adjuvant when the polymer concentration was fixed at 2000 or 4000 μg mL^–1^, respectively. At the higher polymer concentration of 4000 μg mL^–1^, there was no obvious difference in the anti-HBsAg IgG titer between the HBsAg10-PP4000 and HBsAg5-PP4000 groups. This is because with the aid of a sufficiently high concentration of the PEG–PAEEP adjuvant, even a four times lower concentration of antigen (5 μg mL^–1^) could induce a comparable level of antibody expression as shown in Figure 5B. In comparison, HBsAg10-PP2000 led to a 1 magnitude higher antibody concentration than HBsAg5-PP2000 (*p* < 0.01). This indicates that, with the insufficient polymeric adjuvant, the antigen concentration would have a more decisive effect on the immune responses. By comparing HBsAg10-PP4000 with HBsAg10-PP2000, it is also noteworthy that at the relatively higher antigen concentration of 10 μg mL^–1^, the serum IgG titer could be easily increased with a relatively lower adjuvant concentration, which is consistent with the result shown in Figure 5B for HBsAg20-PP4000 and HBsAg20-PP2000. On the other hand, Figure 5C displays that the HBsAg20 control group without the polymeric adjuvant resulted in a significantly lower level of IgG titer than the HBsAg10-PP4000, HBsAg5-PP4000, and HBsAg10-PP2000 groups. It can be inferred that given their lower antigen concentrations, the immune response of HBsAg5 or HBsAg10 alone would not exceed that of HBsAg20 alone. This further proved the adjuvant efficacy of the cationic PEG–PAEEP polymer.

Furthermore, the HBsAg-VLP/PEG–PAEEP formulations with a fixed antigen-to-polymer mass ratio of 100 were investigated (Figure 5D). Increasing antigen concentration during formulation from 5 μg mL^–1^ (HBsAg5-PP500) to 10 μg mL^–1^ (HBsAg10-PP1000) resulted in a considerable increase in IgG titers by 26.7-fold. This enhancement leveled off with further increasing the antigen concentration to 20 μg mL^–1^ (HBsAg20-PP2000).

The HBsAg20-Al500 vaccine formulation (with 20 μg mL^–1^ HBsAg-VLP and 500 μg mL^–1^ aluminum adjuvant) was then used as a positive control to compare the *in vivo* performance of PEG–PAEEP with that of the widely used, commercially available aluminum adjuvant. As shown in Figure 5E, it is encouraging that HBsAg20-PP4000 induced the anti-HBsAg IgG at a similar level as compared with HBsAg20-Al500, while significantly higher than that of HBsAg-VLP alone without adjuvant. Given that no statistically significant increase in the immune responses was observed when the polymer concentration was raised from 2000 to 4000 μg mL^–1^ (Figure 5B) and that HBsAg20-PP4000 carried an equivalent amount of positive charges to the HBsAg20-Al500 positive control, the HBsAg20-PP4000 nanoparticle was chosen as an optimal vaccine formulation for comparison with the positive control in this study. However, it is notable that the antigen and polymeric adjuvant concentrations used in the HBsAg20-PP4000 formulation led to a state of immune response saturation, as presented in Figure 5B–D. A comparative analysis of IgG titer levels using the one-way ANOVA test showed that, despite having lower antigen doses, the HBsAg10-PP4000, HBsAg5-PP4000, and HBsAg10-PP2000 formulations exhibited no significant difference from HBsAg20-Al500 (Figure S2), indicative of the dose-sparing effect of the cationic PEG–PAEEP adjuvant.

Figure S3 shows no significant difference in serum IFN-γ secretion among all of the groups tested, indicating that the immune responses elicited by the PEG–PAEEP adjuvant could be biased toward Th2-type responses. Furthermore, the body weights of mice were monitored during the immunization. No decrease in body weight was observed for all of these HBsAg-VLP/PEG–PAEEP vaccine formulations, indicative of a good *in vivo* tolerability (Figure S4). These results show that PEG–PAEEP can effectively increase the vaccine immunogenicity and has an antigen-dose sparing effect. The antigen concentration, polymer concentration, and antigen-to-polymer mass ratio can be readily optimized to maximize the immunogenicity. This work presents a promising adjuvant for HBsAg-VLP.

## 4 Conclusions

In summary, a novel, biocompatible, and biodegradable PEG–PAEEP polymer was successfully developed as an adjuvant for potentiating the immune responses of the HBsAg-VLP vaccine. The cationic polymer with pendent ethanolamine groups was synthesized by ring-opening polymerization. The HBsAg-VLP/PEG–PAEEP vaccine formulations were easily prepared by mixing HBsAg-VLP and PEG–PAEEP. The stable nanoparticles were formed due to the electrostatic interactions between HBsAg-VLP and PEG–PAEEP. As compared with HBsAg-VLP alone, the HBsAg-VLP/PEG–PAEEP nanoformulations had a larger size and a net positive surface charge, enabling the significantly enhanced cellular uptake by DCs and macrophages. The optimized HBsAg20-PP4000 formulation remarkably improved the anti-HBsAg IgG titer in mice compared to HBsAg-VLP alone after a second immunization. The PEG–PAEEP polymeric adjuvant can effectively increase the vaccine immunogenicity and has an antigen-dose sparing effect, while exhibiting favorable biocompatibility and *in vivo* tolerability profiles. Therefore, the PEG–PAEEP polymer has been demonstrated for the first time as a promising HBsAg-VLP vaccine adjuvant and can be a potential alternative to the commercial aluminum adjuvant.

## Supporting Information Available

Relative hemolysis of RBCs incubated with PEG–PAEEP at different polymer concentrations and for different durations, and body weights of mice during immunization IM with various vaccine formulations (PDF)
