# Supplementary material for: Potentiating the Immune Responses of HBsAg-VLP Vaccine Using a Polyphosphoester-Based Cationic Polymer Adjuvant
Source: ACS Appl Mater Interfaces. 2023 Oct 10;15(42):48871–81. doi: 10.1021/acsami.3c07491 (PMC10614196; doi:10.1021/acsami.3c07491)
Supplement: Supplementary file 1 — am3c07491_si_001.pdf [file am3c07491_si_001.pdf]

Supporting information

Potentiating the immune responses of HBsAg-VLP vaccine using a polyphosphoester-based cationic polymer adjuvant

Xuhan Liu[1,2], Yifan Liu[1], Xiaoyu Yang[3], Xinyu Lu[1], Xiao-Ning Xu[4], Jiancheng Zhang[3], Rongjun Chen[*, 1]

[1] Department of Chemical Engineering, Imperial College London, South Kensington Campus, London SW7 2AZ, UK

[2] Department of Emergency Medicine, Shenzhen University General Hospital, Shenzhen University, Shenzhen 518051, China

[3] AIM Honesty Biopharmaceutical Co., Ltd, Dalian 116620, China

[4] Department of Infectious Diseases, Imperial College London, London W12 0NN, UK

* Corresponding author:

Email: rongjun.chen@imperial.ac.uk

Tel: +44 (0)20 7594 2070

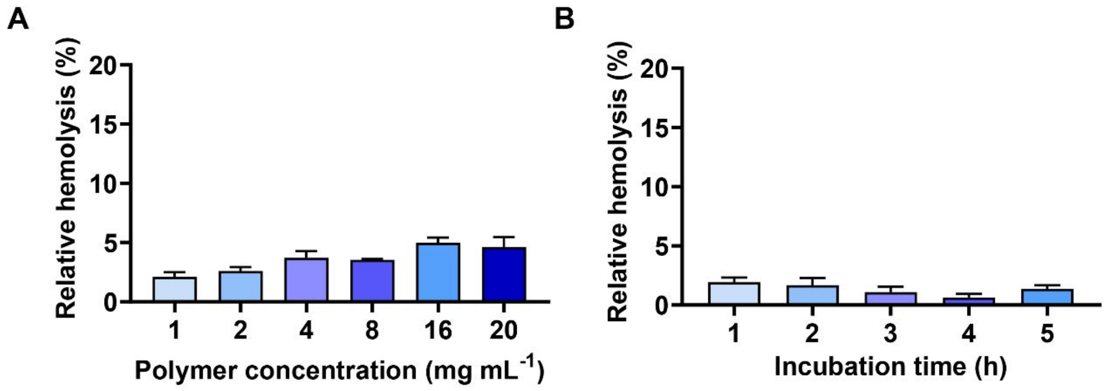

Figure S1. Relative hemolysis of RBCs incubated with PEG-PAEEP (A) at different polymer concentrations for 1 h, and (B) at a fixed polymer concentration of 4 mg mL^{-1} for different durations of time.

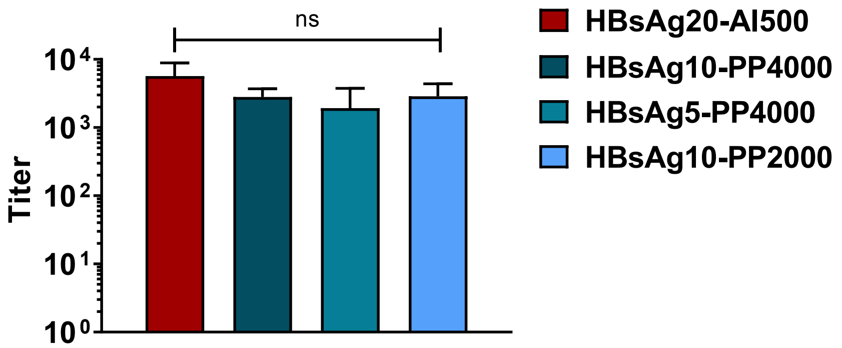

Figure S2. Serum anti-HBsAg IgG titers of the female BALB/c mice immunized intramuscularly with various vaccine formulations at the fixed injection volume of 100 μL. Statistical analysis was performed using the one-way ANOVA test. ns represents no significant difference between two groups.

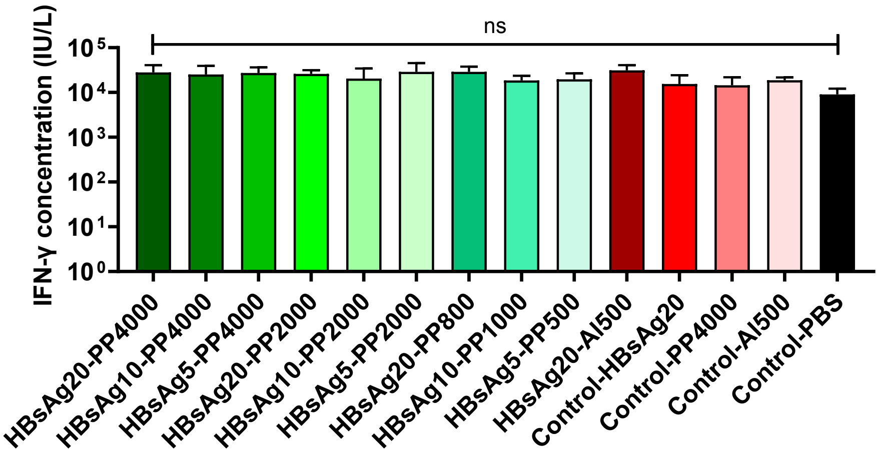

Figure S3. Serum IFN-γ secretion in the female BALB/c mice following the boost immunization intramuscularly with various vaccine formulations at the fixed injection volume of 100 μL. Statistical analysis was performed using the one-way ANOVA test. ns represents no significant difference between two groups.

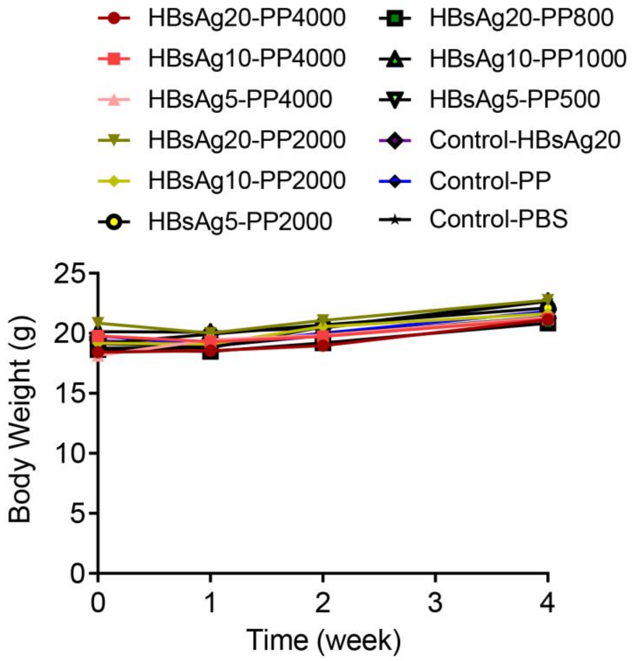

Figure S4. Body weights of the female BALB/c mice during immunization intramuscularly with various vaccine formulations at the fixed injection volume of 100 μL.
